# Patenting and patent challenges in South Korea after introducing a patent linkage system

**DOI:** 10.1186/s12992-022-00887-5

**Published:** 2022-11-12

**Authors:** Kyung-Bok Son

**Affiliations:** College of Pharmacy, Hanyang University, 55 Hanyangdaehak-ro, Sangnok-gu, Ansan-Si, 15588 Gyeonggi-do South Korea

**Keywords:** Access to medicines, Patenting, Patent challenges, Free trade agreements, South Korea

## Abstract

**Background:**

South Korea introduced the patent linkage system in 2015 as part of the implementation of free trade agreements with the United States. This study assessed trends in brand-name drug patenting and generic patent challenges in South Korea after the introduction of the system.

**Methods:**

From 2012–19, we constructed a novel dataset that combines information about listed patents with their corresponding brand-name drugs and patent challenges against these brand-name drugs. We analyzed brand-name drug patenting and generic patent challenges and elucidated factors in timely patent challenges using event history analysis.

**Results:**

During the study period, 659 brand-name drugs listed their patents in the K-Orange Book and patent challenges against 95 brand-name drugs were initiated. The number of listed patents and their nominal patent term varied by the characteristics of the brand-name drugs. Patent challenges of generic drugs were marginal in South Korea even though the surge of patent challenges of generics were noticed right after the introduction of the patent linkage system.

**Conclusions:**

Patenting and patent challenges are critical factors when introducing generic drugs into the market under the patent linkage system. However, the impact of the patent linkage system on patenting and patent challenges could be varied by the specific form of the patent linkage system and the contexts of pharmaceutical markets.

**Supplementary Information:**

The online version contains supplementary material available at 10.1186/s12992-022-00887-5.

## Introduction

Patent linkage refers to the establishment of a system that connects marketing approval for a generic drug and patent status of its reference brand-name drug [1]. Under the system, a generic drug cannot obtain marketing approval when patent litigation is initiated by a marketing holder and/or patent holder of the brand-name drug [2]. A generic drug manufacturer must successfully litigate relevant patents to be granted market approval for a generic drug. Agreements on trade-related aspects of intellectual property rights (TRIPS), which are multilateral trade agreements that set global standards for intellectual property regulations for members of the World Trade Organization (WTO), do not specify a patent linkage system [3]. However, recently signed bilateral/multilateral trade agreements include provisions related to a patent linkage [4]. The Comprehensive and Progressive Agreement for Trans-Pacific Partnership (CPTPP) requires eleven member countries to establish a patent linkage system [5].

The United States first established a patent linkage system in 1984 through the Drug Price Competition & Term Restoration Act of 1984, notably known as “the Hatch–Waxman Act” [6, 7]. The prototypic form of the patent linkage system in the United States had four parts: a patent register, a notification, a stay of approval, and the first generic exclusivity [2, 6]. When a brand-name manufacturer submits a new drug application (NDA) to the regulatory authority, the manufacturer may list relevant patents on the patent register, the so-called “Orange Book” in the United States. An applicant seeking marketing approval for a corresponding generic drug must provide a certification to the regulatory authority and a notification to the brand-name manufacturer contending that the marketing approval for a generic drug will not infringe on patents on the register. The notification may cause an action for infringement by a brand-name manufacturer. This action triggers a stay of marketing approval for a generic drug. To promote patent challenges and timely entrance of a generic drug, market exclusivity is granted to the generic manufacturer who obtained the first marketing approval for the generic drug with successful patent challenges.

Canada, Australia, and South Korea introduced the patent linkage system in 1993, 2005, and 2015, respectively, as part of the implementation of their free trade agreements with the United States [8–10]. Provisions related to patent linkage described in these trade agreements appear to have a degree of constructive ambiguity [4]. Thus, the patent linkage system in these countries offers some flexibilities and comes in different forms [2, 10]. Australia did not adopt the patent register or first generic exclusivity. Canada also did not implement the first generic exclusivity. Despite these variations, patenting and patent challenges are critical factors when introducing generic drugs into the market under the patent linkage system [11]. Many researchers have investigated patenting and patent challenges in the United States [12–16]. Yet empirical evidence regarding the patent linkage system outside the United States is lacking [8]. The patent linkage system in South Korea has reached maturity since its implementation in 2015, providing a valuable opportunity to empirically investigate how patenting and patent challenges are intertwined under the patent linkage system [11]. A case from South Korea may give an exemplar for other countries that contemplating the system in the near future [17, 18].

We assessed trends in brand-name drugs’ patenting and generics’ patent challenges in South Korea after the introduction of the patent linkage system, under which patent challenges are deemed as essential parts of granting timely market approval for generic drugs. To this end, we constructed a novel dataset that combines information about brand-name drugs with their patents and patent challenges against these brand-name drugs. With this novel dataset, we identified brand-name drugs that listed their patents in the K-Orange Book, which is similar to the Orange Book in the United States, presented brand-name drugs’ patenting and generics’ patent challenges, and elucidated factors in timely patent challenges of generic manufacturers.

## Materials and methods

### Study design

This study used several approaches to present patenting and patent challenges in South Korea after the introduction of the patent linkage system. We retrieved patents in the K-Orange Book and identified their corresponding brand-name drugs. The number of patents listed in the K-Orange Book and the nominal patent term were used to present patenting behavior of brand-name drugs manufacturers. Nominal patent term was defined as a year difference between the date of granting market approval and the date of the last expiring patent. We also combined information on patent challenges against these brand-name drugs.

### Data source

We examined patents listed in the K-Orange Book and then identified their corresponding brand-name drugs. Patents listed on K-Orange Book are subjects of the patent linkage system. K-Orange Book is publicly accessible and searchable at the website of the Ministry of Food and Drug Safety (MFDS) [19]. For each drug, we collected information about drug types (chemical or biologic), statutory exclusivity, manufacturer types (domestic or foreign manufacturer), route of administration (oral, injectable, or other forms), and anatomical therapeutic chemical (ATC) classification from the MFDS website. Statutory exclusivity indicates data exclusivity granted for new drugs, new administration, and new indication. During this period, a generic drug manufacturer could not use safety and efficacy data that have been submitted for granting marketing approval for a brand-name drug [20]. Information about the market size and number of manufacturers at the market were obtained from the Health Insurance Review and Assessment Service (HIRA) [21]. For these two variables, the market was defined according to the third category of the ATC classification. Information about patent challenges was also retrieved from the MFDS website [22].

### Analytical methods

We presented brand-name drugs’ patenting, including the number of listed patents and nominal patent term per drug, and generics’ patent challenges. For patent challenges, we were interested in the timing of patent challenges. Our observations were right-censored, implying that some patent challenges could be initiated by generic drugs manufacturers in the foreseeable future. Thus, we adopted an event history model for a statistical estimation of the duration. Supplementary File 1 describes variables used in this study. Duration was defined as a year difference between the date of market approval for a brand-name drug and the date of the first patent challenge. Drug type, statutory exclusivity, route of administration, manufacturer type, number of patents, market size, and number of manufactures at the market were added as independent variables.

Kaplan–Meier survival estimates were used to describe different trends among selected variables. The Cox proportional hazards model was then adopted to determine the relative impact of variables on the duration [23, 24]. Proportional hazards assumption is a key assumption of the Cox model. In particular, the Cox model assumes that each covariate has a multiplicative effect in the hazards function and that this multiplicative effect is constant over time. However, the proportional hazards assumption could not be applied in some circumstances. We used two validation methods for the assumption: a graphical approach using log minus log plot and a goodness of fit test. We found that some variables such as approval year and ATC classification violated the proportional hazards assumption. Thus, we applied a stratified Cox proportional hazard model to control variables that violated the proportional hazards assumption [23]. Data management and analysis were performed using R statistical software (version 3.4.3). Statistical significance was assumed when the *p*-value was less than 0.05.

## Results

### Investigated drugs

Table 1 describes characteristics of investigated drugs sorted by approval year. During the study period, 659 brand-name drugs listed their patents in the K-Orange Book. Of these 659 drugs, 555 (84%) were chemical entities, 442 (67%) were in oral forms, 431 (65%) were granted market approval by foreign manufacturers, and 402 (61%) were granted statutory exclusivity. The number of drugs sorted by their approval year ranged from 36 in 2018 to 112 in 2015. In 2012, 68 brand-name drugs were approved. Of these 68 drugs, 60 (88%) were chemical entities, 46 (68%) were granted market approval by foreign manufacturers, 43 (63%) were in oral forms, and 40 (59%) were granted statutory exclusivity. In contrast, 48 brand-name drugs were approved in 2019. Of these 48 drugs, 37 (77%) were chemical entities, 32 (67%) were granted market approval by foreign manufacturers; 30 (63%) were in oral forms, and 40 (83%) were granted statutory exclusivity.

**Table 1:** Characteristics of the investigated drugs sorted by approval year

Approval year	2012	2013	2014	2015	2016	2017	2018	2019	Total
Number of drugs (N)	68	100	107	112	99	89	36	48	659
Drug type									
Chemical	60	90	86	89	90	74	29	37	555
Biologic	8	10	21	23	9	15	7	11	104
Statutory exclusivity									
No	28	55	46	48	27	40	5	8	257
Yes	40	45	61	64	72	49	31	40	402
Manufacturer type									
Domestic	22	44	27	37	35	39	8	16	228
Foreign	46	56	80	75	64	50	28	32	431
Administration									
Oral	43	76	71	66	72	59	25	30	442
Injection	16	16	26	30	16	24	8	11	147
Others	9	8	10	16	11	6	3	7	70
ATC classification									
J/L	19	18	30	40	27	33	14	13	194
A/B/C	31	43	36	43	47	35	14	9	258
M/N	6	18	28	15	5	5	3	18	98
Others	12	21	13	14	20	16	5	8	109

### Patenting and patent challenges

Table 2 presents the number of patents listed in the K-Orange Book per drug sorted by approval year. The mean value ranged from 1.69 in 2018 to 2.10 in 2013. We analyzed the number of patents according to the characteristics of drugs. Interestingly, biologics had more patents than chemical drugs. During the observation period, the mean number of patents of biologics was 2.08, while that of chemical drugs was 1.84. In a similar vein, drugs introduced by foreign manufacturers had more patents than drugs introduced by domestic manufacturers (2.17 versus 1.33).

**Table 2:** The number of patents listed in the K-Orange Book per drug sorted by approval year

Approval year	2012	2013	2014	2015	2016	2017	2018	2019	Total
Number of drugs (N)	68	100	107	112	99	89	36	48	659
Mean	1.72	2.10	1.90	1.80	1.85	1.84	1.69	2.00	1.87
Standard deviation	1.26	1.61	1.09	0.94	1.04	1.52	0.74	1.36	1.24
Drug type									
Chemical	1.77	2.01	1.92	1.78	1.88	1.65	1.83	1.84	1.84
Biologic	1.37	2.90	1.86	1.91	1.67	2.80	1.14	2.55	2.08
Statutory exclusivity									
No	1.79	2.11	1,78	1.58	1.59	1.55	1.40	2.00	1.76
Yes	1.68	2.09	2.00	1.97	1.96	2.08	1.74	2.00	1.96
Manufacturer type									
Domestic	1.31	1.27	1.11	1.38	1.43	1.51	1.38	1.12	1.33
Foreign	1.91	2.75	2.17	2.01	2.09	2.10	1.79	2.44	2.17
Administration									
Oral	1.91	2.05	2.01	1.88	1.89	1.68	1.76	1.80	1.90
Injection	1.38	2.56	1.85	1.93	1.81	2.29	1.25	2.55	1.98
Others	1.44	1.62	1.30	1.25	1.73	1.67	2.33	2.00	1.56
ATC classification									
J/L	1.58	3.22	2.03	2.15	1.96	2.15	1.71	3.00	2.18
A/B/C	1.90	1.40	1.81	1.65	1.79	1.80	1.71	2.11	1.72
M/N	1.67	2.39	2.07	1.87	1.80	1.40	1.00	1.28	1.85
Other	1.50	2.33	1.54	1.21	1.90	1.44	2.00	1.88	1.74

**Fig. 1:**
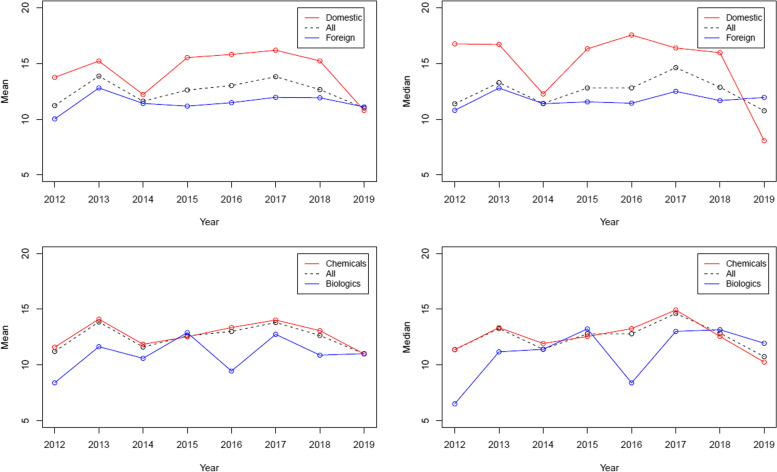
Nominal patent term sorted by approval year

Figure 1 presents mean and median years of nominal patent term grouped by manufacturer and drug types. The first row in Fig. 1 describes mean and median nominal patent term grouped by domestic and foreign manufacturers, respectively. The nominal patent term of drugs introduced by domestic manufacturers was longer than that of drugs introduced by foreign manufacturers. The second row in Fig. 1 describes mean and median nominal patent terms grouped by chemical entities and biologics, respectively. The nominal patent term of chemical entities was longer than that of biologics.

Table 3 describes characteristics of the drugs under patent challenges sorted by challenge year. During the study period, patent challenges against 95 brand-name drugs were identified. Of these 95 brand-name drugs, 94 (99%) were chemical entities, 83 (87%) were in oral forms, 75 (79%) were granted market approval by foreign manufacturers, 74 (78%) were granted statutory exclusivity by the MFDS, and 52 (55%) belonged to A/B/C according to the first category of the ATC classification system. When we sorted these brand-name drugs by their challenged year, 63 (66%) brand-name drugs were challenged in 2015, the year when the patent linkage system was introduced.

**Table 3:** Characteristics of drugs under patent challenges sorted by challenge year

Challenge year	2007	2012	2013	2014	2015	2016	2017	2018	Total
Number of drugs (N)	1	2	6	1	63	11	6	5	95
Drug type									
Chemical	1	2	6	0	63	11	6	5	94
Biologic	0	0	0	1	0	0	0	0	1
Statutory exclusivity									
No	1	1	2	0	5	7	4	1	21
Yes	0	1	4	1	58	4	2	4	74
Manufacturer type									
Domestic	1	1	4	0	9	3	1	1	20
Foreign	0	1	2	1	54	8	5	4	75
Administration									
Oral	1	1	6	0	55	10	6	4	83
Injection	0	0	0	1	2	0	0	1	4
Others	0	1	0	0	6	1	0	0	8
ATC classification									
J/L	0	0	2	1	9	1	0	0	13
A/B/C	0	0	3	0	37	4	4	4	52
M/N	1	1	0	0	6	4	0	0	12
Others	0	1	1	0	11	2	2	1	18

Table 4 presents characteristics of the drugs sorted by experiencing patent challenges. In this table, we excluded brand-name drugs approved in 2018 and 2019 to guarantee an observation period of at least three years. Of 575 brand-name drugs, 94 (16%) drugs were challenged by generic manufacturers. The proportion of brand-name drugs challenged by generic manufacturers varied by characteristics of brand-name drugs. Chemical drugs (19%), drugs with statutory exclusivity (22%), drugs introduced by foreign manufacturers (20%), and drugs in oral forms (21%) presented higher likelihood of being challenged. In contrast, biologics (1%), drugs without statutory exclusivity (9%), drugs introduced by domestic manufacturers (9%), and drugs in injection form (3%) presented a lower likelihood of being challenged. We also found that the number of patents listed in the K-Orange Book was significantly different between the non-challenged and challenged group. However, market size and number of manufacturers at the market grouped according to the third category of the ATC classification were not significantly different between the two groups.

**Table 4:** Characteristics of the drugs sorted by patent challenges

	Non-challenged	Challenged	% of challenged	*P*-value
Number of drugs (N)	481	94	(16%)	
Drug type				< 0.0001
Chemical	396	93	(19%)	
Biologic	85	1	(1%)	
Statutory exclusivity				< 0.0001
No	223	21	(9%)	
Yes	258	73	(22%)	
Manufacturer type				0.0010
Domestic	185	19	(9%)	
Foreign	296	75	(20%)	
Administration				< 0.0001
Oral	305	82	(21%)	
Injection	124	4	(3%)	
Others	52	8	(13%)	
ATC classification				0.0025
J/L	154	13	(8%)	
A/B/C	184	51	(22%)	
M/N	65	12	(16%)	
Others	78	18	(19%)	
Year				< 0.0001
2012	44	24	(35%)	
2013	82	18	(18%)	
2014	82	25	(23%)	
2015	98	14	(13%)	
2016	92	7	(7%)	
2017	83	6	(7%)	
Patents	1.79	2.34		0.0042
Market size	413,749	466,502		0.1136
Number of manufacturers	379	395		0.5841

Drugs approved from 2018 to 2019 were excluded in order to guarantee an observation period of at least three years

### Timing of patent challenges

Supplementary File 2 provides a descriptive overview of the duration using the Kaplan–Meier curve. The duration was measured in the year between the date of marketing approval of the drug and the date of patent challenge against the drug. The vertical axis of the figure indicates the conditional probability that the patent challenge will occur after a given period. Specifically, the first curve in Supplementary File 2 showed that 80% of brand-name chemical drugs remained unchallenged after eight years from the date of their marketing approval. In contrast, few biologics (1 out of 86 biologics) experienced patent challenge after 8 years from the date of their marketing approval. Supplementary File 3 presents log minus log plot of Kaplan–Meier estimation with log-rank test between variables. In this plot, we found that variables on the ATC classification and the year of market approval might violate the proportional hazards assumption. In a similar vein, we found that the year of market approval might violate the assumption when we conducted the goodness of fit test. Thus, we fitted the stratified Cox model with four discrete factors (drug types, statutory exclusivity, manufacturer types, and route of administration) and three continuous factors (patents, market size, and number of manufacturers).

Table 5 provides results from the stratified Cox model estimation. Drugs with a statutory exclusivity, drugs introduced by foreign manufacturers, drugs belonging to big markets, and drugs with increased number of listed patents presented a short time to patent challenges. In contrast, biologics presented a delayed time to patent challenges. Other variables, including administration types and number of manufacturers in the market, did not presented significant effect on the timing of patent challenges.

**Table 5:** Results from the stratified Cox proportional hazard model estimation

	Coefficient	Standard Error	*P*-value
Drug type: Biologic (Ref. Chemical)	-3.17	1.24	0.0105
Statutory exclusivity: Yes (Ref. No)	1.25	0.29	< 0.0001
Manufacturer type: Foreign (Ref. Domestic)	1.14	0.30	0.0001
Administration: Injection (Ref. Oral)	-0.41	0.63	0.5177
Administration: Others (Ref. Oral)	-0.33	0.51	0.5270
Patents	0.48	0.23	0.0389
Market size	0.65	0.29	0.0284
Number of manufacturers	-0.40	0.24	0.0999

## Discussion

South Korea introduced the patent linkage system in 2015 as part of the implementation of free trade agreements with the United States. Under the patent linkage system, patenting and patent challenges are critical factors when introducing generic drugs into the market. This study assessed trends in brand-name drugs’ patenting and generics’ patent challenges in South Korea after the introduction of the patent linkage system.

### Patenting of brand-name drugs

In the United States, an increased number of patents and a prolonged nominal patent term of brand-name drugs were observed after the introduction of the patent linkage system [25–27]. In contrast, some interesting trends in the behavior of brand-name drug manufacturers were noted in South Korea. First, the number of brand-name drugs listed their patents in the K-Orange Book did not increase during the study period. The mean number of brand-name drugs approved from 2012–2015 was 91.6, whereas that of brand-name drugs approved from 2016–2019 was 69.0. Second, the mean number of patents listed in the K-Orange Book per brand-name drug slightly increased during the study period. Mean numbers of patents per brand-name drug approved in 2012 and 2019 were 1.72 and 2.00, respectively. The nominal patent term sorted by the approval year was maintained during the study period.

These findings could be explained by characteristics of the patent linkage system in South Korea [2, 9]. In the United States, manufacturers may list additional patents in the Orange Book after market approval of the brand-name drug. However, patents granted after market approval were not eligible to be listed in the K-Orange Book in South Korea. Furthermore, the timing of patent information submission to be listed in the K-Orange Book is specified within 30 days of the date of market approval. In a similar vein, K-Orange Book is managed with audit and examination by the MFDS [2, 9]. Sometimes, the regulatory authority deletes irrelevant patents in the list. It is noteworthy that the US authority could not amend or delete patents in the Orange Book on its own.

Although patenting of brand-name drugs did not increase in South Korea, some findings help explain the behavior of brand-name manufacturers. The patenting was related to the characteristics of brand-name drugs. The number of patents was larger for biologics and drugs introduced by foreign manufacturers compared to that of chemicals and drugs introduced by domestic manufacturers. Thus, we could expect that the nominal patent term of these drugs would be longer than their counterparts. However, the nominal patent term of biologics and drugs introduced by foreign manufacturers was shorter than that of their counterparts. The difference in the nominal patent term indicated variations in the developing and/or patenting strategy of brand-name drug manufacturers. The nominal patent term was measured by a year difference between the date of marketing approval and the date of the last expiring patent. The date of the last expiring patent was associated with the filing date of the patent, implying that the manufacturer introducing biologics and foreign manufacturers filed the patent earlier than did their counterparts.

### Surge in patent challenges of generic drugs

Generics’ patent challenge is another interesting topic under the patent linkage system [28, 29]. We found that the patent linkage system caused a surge of patent challenges in South Korea. Brand-name drugs challenged by generic manufacturers were rare in South Korea before 2015, when 9-month exclusivity under the patent linkage system was introduced. However, 63 brand-name drugs were challenged by generic manufacturers in 2015, implying that the patent linkage system caused a surge of patent challenges. Generic manufacturers waited to obtain 9-month exclusivity as the reward for a successful patent challenges. In a similar vein, types of drugs challenged by generic manufacturers were expanded with the patent linkage system. Before 2015, drugs in injection form and drugs with statutory exclusivity were not the main subjects of patent challenges. However, some patent challenges against these drugs occurred after introducing the system.

Despite the surge in patent challenges right after the introduction of the patent linkage system, patent challenges in South Korea are still less frequent than in other countries. We calculated the portion of brand-name drugs that experienced patent challenges. Of 575 brand-name drugs approved from 2012–17, only 94 (16%) were challenged by generic manufacturers. In other words, 15.6 brand-name drugs per year experienced patent challenges in South Korea. The US FDA has released information about the “Paragraph IV challenge.” In the United States, the number of brand-name drugs experiencing challenges ranged from 52–65 per year for the same study period (2012–17) [30]. Health Canada annually updated information on “Notice of Allegation,” similar to “Paragraph IV challenges” in the United States. For the same study period, the number of brand-name drugs that received “Notice of Allegation” ranged from 105–176 per year in Canada [31].

### Factors affecting timing of patent challenges

We applied an event history model to elucidate factors in patent challenges. With this empirical approach, we confirmed that additional patenting was associated with timely patent challenges. Patents for an active ingredient are positioned at high hierarchies, whereas patents for formulation are positioned at low hierarchies [32]. We could not identify the characteristics of patents in this study. However, it is reasonable to assume that additional patenting is associated with patents at low hierarchies. Given this, we could conclude that challenges against patents at low hierarchies are initiated by generic manufacturers in a timely manner in South Korea. In addition to patent hierarchies within brand-name drugs, we found patent hierarchies between brand-name drugs. Patents for biologics and injectable drugs were not likely to be the subjects of patent challenges. However, patents for chemicals and drugs with statutory exclusivity were more likely to be subjects of such challenges. Patent challenges against biologics and/or the entrance of biosimilars into the market is a debatable issue in pharmaceutical policy [33, 34]. We confirmed that biologics had more patents than chemical drugs. However, biologics were less likely to be challenged by generic manufacturers in a timely manner.

As demonstrated in this study, patents at lower hierarchies caused patent challenges by generic manufacturers. The question becomes, why do so many brand-name manufacturers list their weak patents in the K-Orange Book? Researchers explained that even patents at lower hierarchies could make the patent portfolio stronger and that a patent portfolio could discourage generic competition [14]. They become additional barriers for generic manufacturers to enter the market. Defeating both patents is required for generic manufacturers to introduce generics to the market. Generic manufacturers should search for, evaluate, and challenge both patents and obtain a favorable decision that both patents are invalid and/or not infringed on in court. Furthermore, the patent linkage system requires generic manufacturers to notify brand-name manufacturers that they will introduce generics contending that the patent is invalid and/or not being infringed. Thus, brand-name manufacturers could cope with patent challenges more effectively.

We found that increased market size was an abbreviating factor in patent challenges, implying that manufacturers’ expectation of profits might play a role in the decision-making for a patent challenge. The result from this empirical approach is in line with the literature. A questionnaire survey was conducted to identify factors encouraging patent challenge in South Korea [35]. Employees of domestic manufacturers responded that market size, expectations for winning the trial, and the possibility of market approval were the most influential factors in deciding patent challenges. These results shed light on the rarity of patent challenges in South Korea. The limited number of patent challenges might be associated with manufacturers’ low expectation of profit when they market generics. The market share of generics is small in South Korea, even though the price of generics is comparatively high [36]. More specifically, the market share of generics based on volume and value in South Korea has been reported to be lower than that in the United States or Canada [37]. In a similar vein, employees at domestic manufacturers argued that the economic incentive incurred by initiating patent challenge was smaller than the economic disincentive incurred by not initiating a patent challenge [35].

### Limitations

This study has several limitations. First, this study investigated brand-name drugs that listed their patents in the K-Orange Book. Brand-name drugs might not list their patents in the register, implying that patenting and patent challenges outside the patent linkage system were not included in this study. Second, this study could not identify patents according to their hierarchies. In South Korea, patents on drug substance, composition, dosage form, and pharmaceutical use are eligible to be listed. Identifying and categorizing these patents might provide a valuable opportunity to fully understand the effect of the system. Third, as abovementioned, provisions related to patent linkage have a degree of ambiguity, and the patent linkage systems in selected countries have variations [2]. Thus, results from this study are not currently generalizable to other countries. However, experience from South Korea could shed light on understanding the impact of the patent linkage system on patenting and patent challenges.

## Conclusions

Patenting and patent challenges are critical factors when introducing generic drugs into the market under the patent linkage system. Compared to the United States, patenting of brand-name drugs and patent challenges of generic drugs were marginal in South Korea, even though a surge of patent challenges of generic drugs was noticed right after the introduction. The impact of the patent linkage system on patenting and patent challenges could be varied by the specific form of the patent linkage system and the contexts of pharmaceutical markets. The provisions related to patent linkage have a degree of ambiguity, implying that contemplating the system has some flexibility. Limiting patents that can be listed and regular audit or examination of the list by the authorities could prevent unnecessary patenting of brand-name drugs. An increased market shares of generics and/or manufacturers’ high expectation of profit when they introduce generics into the market could encourage patent challenges.

## Supplementary Information

**Additional file 1: Supplementary File 1.** Descriptions of the variables.

**Additional file 2: Supplementary File 2.** Kaplan-Meier curve of the selected variables.

A**dditional file 3: Supplementary File 3.** Log minus log plot of Kaplan-Meier estimation with log-rank test between the selected variables.

## Data Availability

The data underlying this article will be shared upon reasonable request to the corresponding author.

## Abbreviations

ATC: Anatomical Therapeutic Chemical
CPTPP: Comprehensive and Progressive Agreement for Trans-Pacific Partnership
HIRA: Health Insurance Review and Assessment Service
MFDS: Ministry of Food and Drug Safety
NDA: New Drug Application
TRIPS: Trade-Related Aspects of Intellectual Property Rights
WTO: World Trade Organization
