# Supplementary material for: Patenting and patent challenges in South Korea after introducing a patent linkage system
Source: Global Health. 2022 Nov 12;18:95. doi: 10.1186/s12992-022-00887-5 (PMC9652859; doi:10.1186/s12992-022-00887-5)
Supplement: Supplementary file 1 — Additional file 1: Supplementary File 1. Descriptions of the variables. [file 12992_2022_887_MOESM1_ESM.docx]

Supplementary File 1. Descriptions of the variables

| Variable | Description | Source |
| --- | --- | --- |
| Duration | The year difference between the date of marketing approval for a brand-name drug and the first patent challenge | MFDS |
| Drug types | Types of the brand-name drug: chemical entity (0), biologics (1) | MFDS |
| Statutory exclusivity | Existence of statutory exclusivity of the brand-name drug granted by the Ministry of Food and Drug Safety: no (0), yes (1) | MFDS |
| Route of administration | Types of administration of the brand-name drug: oral (0), injectable (1), and other forms (2) | MFDS |
| Manufacturer types | Types of manufacturers of the brand-name drug: domestic (0) and foreign manufacturers (1) | MFDS |
| Patents | Number of patents of the brand-name drug listed in the K-Orange Book | MFDS |
| Market size | Sales of drugs on the market grouped according to the third category of the ATC classification in 2017 | HIRA |
| Number of manufacturers | Number of manufacturers at the market grouped according to the third category of the ATC classification in 2017 | HIRA |
