# Supplementary figures and images for: Patenting and patent challenges in South Korea after introducing a patent linkage system
Source: Global Health. 2022 Nov 12;18:95. doi: 10.1186/s12992-022-00887-5 (PMC9652859; doi:10.1186/s12992-022-00887-5)

Supplementary File 2. Kaplan-Meier curve of the selected variables


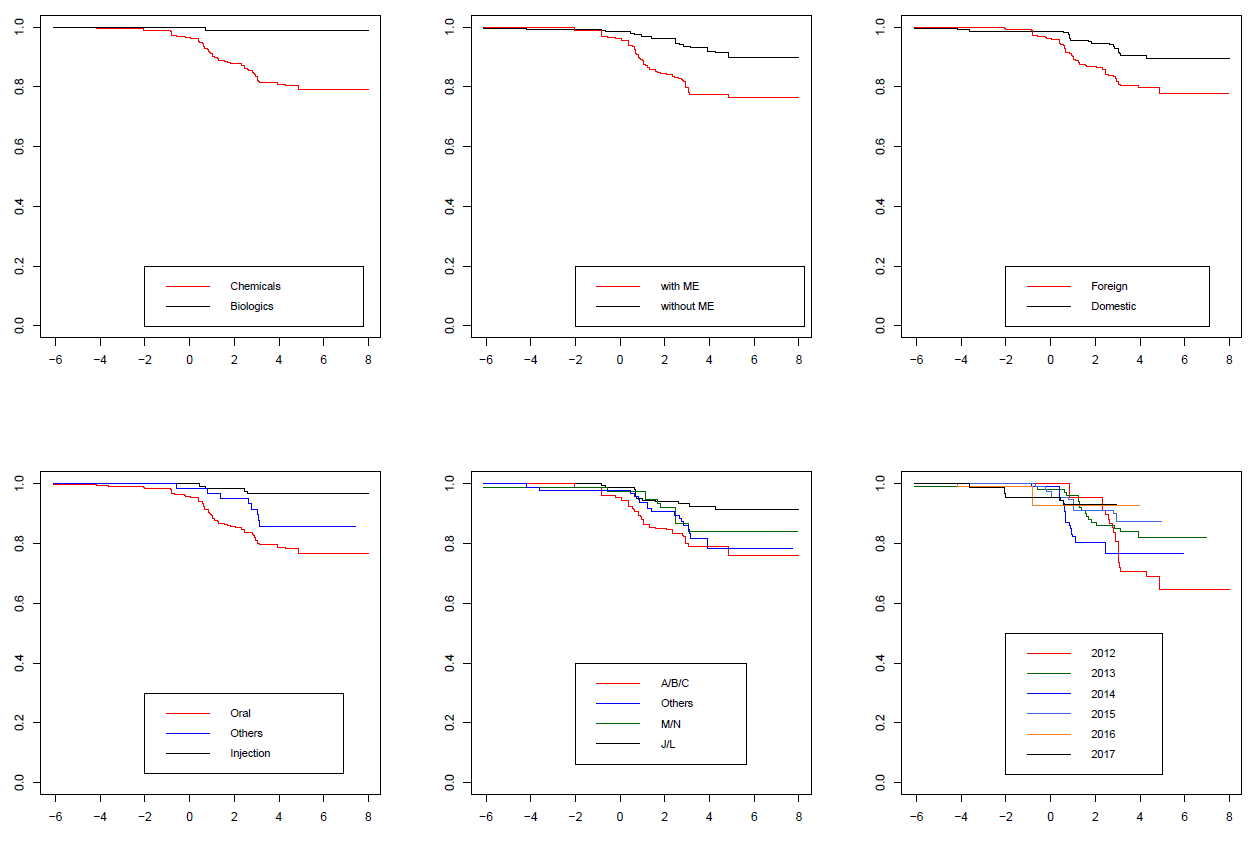

Supplement: Supplementary file 2 — Additional file 2: Supplementary File 2. Kaplan-Meier curve of the selected variables. [file 12992_2022_887_MOESM2_ESM.docx]
