# Supplementary material for: Patenting and patent challenges in South Korea after introducing a patent linkage system
Source: Global Health. 2022 Nov 12;18:95. doi: 10.1186/s12992-022-00887-5 (PMC9652859; doi:10.1186/s12992-022-00887-5)

Supplementary File 3. Log minus log plot of Kaplan-Meier estimation with log-rank test between the selected variables


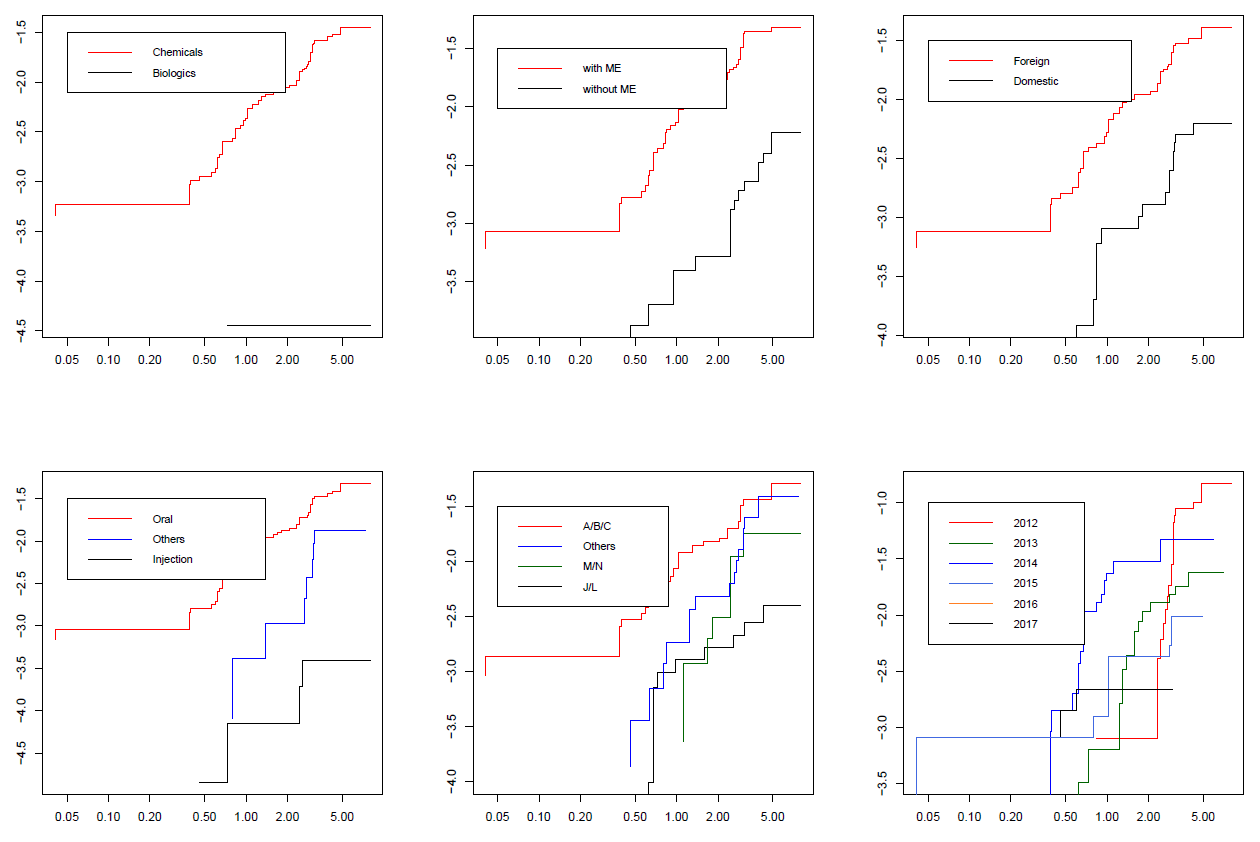

Supplement: Supplementary file 3 — Additional file 3: Supplementary File 3. Log minus log plot of Kaplan-Meier estimation with log-rank test between the selected variables. [file 12992_2022_887_MOESM3_ESM.docx]
